# Bullying, Mental Health, and the Moderating Role of Supportive Adults: A Cross-National Analysis of Adolescents in 45 Countries

**DOI:** 10.3389/ijph.2022.1604264

**Published:** 2022-03-22

**Authors:** Samuel Seunghan Kim, Wendy Marion Craig, Nathan King, Ludwig Bilz, Alina Cosma, Michal Molcho, Gentiana Qirjako, Margarida Gaspar De Matos, Lilly Augustine, Kastytis Šmigelskas, William Pickett

**Affiliations:** ^1^ Department of Psychology, Queen’s University, Kingston, ON, Canada; ^2^ Department of Public Health Sciences, Queen’s University, Kingston, ON, Canada; ^3^ Department of Health Sciences, Brandenburg University of Technology Cottbus-Senftenberg, Senftenberg, Germany; ^4^ Sts Cyril and Methodius Faculty of Theology, Olomouc University Social Health Institute, Palacky University in Olomouc, Olomouc, Czechia; ^5^ Department of Psychology, Babes Bolyai University, Cluj Napoca, Romania; ^6^ Department of Children’s Studies, School of Education, National University of Ireland Galway, Galway, Ireland; ^7^ Institute of Public Health, Tirana, Albania; ^8^ Faculty of Medicine, University of Medicine, Tirana, Albania; ^9^ Aventura Social, Faculty of Human Kinetics, University of Lisbon, Lisbon, Portugal; ^10^ ISAMB, Medicine Faculty, University of Lisbon, Lisbon, Portugal; ^11^ CHILD Research Group, School of Education and Communication, Jönköping University, Jönköping, Sweden; ^12^ Health Research Institute, Faculty of Public Health, Medical Academy, Lithuanian University of Health Sciences, Kaunas, Lithuania; ^13^ Department of Health Psychology, Faculty of Public Health, Medical Academy, Lithuanian University of Health Sciences, Kaunas, Lithuania; ^14^ Department of Health Sciences, Brock University, St. Catharines, ON, Canada

**Keywords:** mental health, HBSC, bullying, cyber-bullying, victimization, adult support, adolescents

## Abstract

**Objectives:** Relationships with supportive adults during adolescence may be a protective factor that lowers the risks associated with bullying. The current study aimed to examine the moderating role of supportive adults in the associations between bullying involvement (in-person and cyber) and mental health problems (psychological symptoms and low life satisfaction).

**Methods:** Data from 45 countries and regions taking part in the 2017/18 Health Behaviour in School-Aged Children study (*N* = 230,757) were used. Multivariable Poisson regression models were used to estimate relative risks of bullying on mental health. Effect estimates were compared across the number of supportive adults to examine a possible cumulative protective effect of relationships with supportive adults.

**Results:** Bullying involvement was consistently associated with poor mental health across the 45 countries. Risk of mental health problems associated with bullying involvement was greatest among students reporting relationships with multiple supportive adults. This was true for all indicators of bullying involvement.

**Conclusion:** Bullying remains a prevalent and harmful experience for youth worldwide. Merely having supportive adults is not sufficient in protecting youth from experiencing the mental health risks associated with bullying.

## Introduction

Bullying is a form of peer aggression in which unwanted aggressive behaviours are perpetuated by youth onto another youth or group of youths, within a context of an observed or perceived power imbalance [1]. Bullying can occur in different forms, including physical, emotional, relational, and even electronically, in the form of cyber-bullying [2]. Previous cross-national studies have found that rates of traditional in-person bullying and victimization vary by country across Europe and North America, ranging from 0.3% to 30% and 0.5% to 32%, respectively [3]. Estimates of cyber-bullying and cyber-victimization also vary, ranging between 0.6% to 31% and 3% to 29%, respectively [3]. Another systematic review found that the prevalence of in-person bullying perpetration and victimization were 34.5% [34.3%–34.8%] and 36% [35.8%–36.2%], respectively [4]. The same study estimated the prevalence of cyber-bullying perpetration to be 15.5% [15.3%–15.7%] and cyber-victimization prevalence of 15.2% [15.1%–15.4%] [4]. Bullying remains a significant public health problem facing youth during a developmental period in which mental health concerns begin to emerge [5].

Traditional in-person bullying perpetration and victimization are both associated with heightened risks of psychosomatic symptoms, such as headaches, stomachaches, irritability, low mood, sleep difficulties, and feeling of helplessness and loneliness [6]. Traditional bullying victimization is also significantly associated with higher risk of suicide attempts among adolescents, as youth who experience bullying at least one time in a month are at 3-fold higher odds of attempting suicide compared to non-bullied adolescents [7]. While there is extensive support for the negative consequences of bullying victimization on adolescent mental health including, lower life satisfaction [8], emotional and behavioural problems [9, 10], and mental disorders in adulthood [11], there is limited evidence regarding the associations between bullying perpetration and mental health problems. Previous research suggests that bullying perpetration is both cross-sectionally and longitudinally associated with internalizing symptoms, including depressed mood, feelings of anxiety, and sleep problems [9]. Furthermore, cyber-bullying perpetration and victimization are both associated with mental health problems [12, 13] and lower life satisfaction [14]. Collectively, research suggests that all forms of bullying take a toll on adolescent mental health, indicating a need for effective prevention and intervention strategies.

Research is needed to identify factors that mitigate the negative effects of bullying involvement on adolescents’ mental health. Examining culturally universal protective factors, such as supportive relationships, can help identify common factors that promote mental wellbeing among youth involved in bullying as either a perpetrator or a victim. Specifically, having supportive relationships with adults (e.g., parent(s), teacher(s)) may be important in lowering the mental health risks associated with bullying perpetration and victimization. Adolescents who report having supportive relationships with parents and teachers are less likely to be involved in bullying at school compared to youth with less adult support, [15–17]. Adults can support adolescents in various ways, such as by demonstrating care and trust (emotional support), spending time with youth (instrumental support), providing information or advice (informational support), or providing evaluative feedback (appraisal support) [18]. Specific to bullying situations, parents and teachers can provide various forms of support to youth involved in bullying, possibly moderating the relationships between bullying experiences and associated mental health outcomes. Adult intervention during bullying situations reduces the psychological harms associated with bullying [19, 20]. When they become aware of bullying, parents and teachers can address youths’ concerns related to safety, alleviate distress, and prevent future bullying incidents. There is evidence that both teachers and parents’ actions and support can buffer (i.e., moderate) against the mental health risks associated with bullying victimization [21, 22] and bullying perpetration [19].

Less is known about how parents and teachers can protect youth against the risks associated with cyber-bullying. Adults tend to perceive cyber-bullying as less serious than in-person bullying [23]. Youth may also be less likely to disclose cyber-bullying to parents and teachers compared to more traditional forms of bullying because they perceive that adults may not take the incident seriously or worsen the situation, or because they perceive adults as unhelpful [24]. Furthermore, since cyber-bullying can occur more covertly, parents and teachers may not be as aware of cyber-bullying compared to in-person bullying [25], and may be less likely to intervene as a result [26, 27]. Adults may not feel technologically competent in addressing cyber-bullying, which occurs through rapidly evolving electronic mediums [28]. For example, teachers may not view cyber-bullying as part of their professional mandate, as it often occurs outside of school [23]. Thus, having supportive adults may be less protective against the mental health risks associated with cyber-bullying involvement compared to traditional forms of bullying.

Research examining factors that mitigate the risks associated with both traditional and cyber-bullying is needed because involvement in cyber-bullying uniquely contributes to adolescents’ mental health, over and above the effects of involvement in traditional bullying [29, 30]. Furthermore, existing research has primarily separately examined how either parent-adolescent and teacher-adolescent relationships attenuate the risks associated with bullying perpetration or victimization, without considering the possible cumulative effects of having supportive adults across multiple settings salient to youth (e.g., home and school). We could expect that there would be a cumulative protective effect of multiple supportive adults (e.g., supportive parent and teacher). Understanding how the association between bullying involvement (i.e., perpetration and victimization) and mental health problems changes as a function of the number of supportive adults in adolescent’s lives can inform bullying intervention efforts to foster healthy social contexts for youth at home and school.

To address these gaps in the literature, the current study aimed to: 1) describe cross-national patterns of in-person bullying perpetration, in-person bullying victimization, cyber-bullying perpetration, and cyber-victimization; 2) describe cross-national patterns in the associations between in-person bullying perpetration, in-person bullying victimization, cyber-bullying perpetration, cyber-victimization, and mental health problems (psychological symptoms and low life satisfaction); and 3) examine the moderating role of supportive relationships with an adult (e.g., parent(s)/teacher(s)) in the associations between in-person bullying perpetration, in-person bullying victimization, cyber-bullying perpetration, cyber-victimization, and mental health problems. We hypothesized that: 1) adolescent experiences of in-person and online bullying and victimization would be associated with increased risks for psychological symptoms and low life satisfaction; 2) the potential negative mental health effects of bullying involvement would be mitigated by relationships with supportive adults; and 3) there would be a dose-response relationship such that as the number of supportive adults increased, the risk of poor mental health associated with bullying would decrease.

## Methods

### Sample and Procedure

This study used cross-sectional data from the 2017/2018 Health Behaviour in School-Aged Children (HBSC) study [31]. The HBSC is a cross-national, school-based child health promotion survey affiliated with the World Health Organization Regional Office for Europe, conducted every 4 years. The 2017/2018 cycle involved 46 countries and regions across Europe, North America, and the Middle East of which 45 were included in the present analysis. National surveys were conducted according to a standard international protocol with student participants nested within schools selected to be nationally representative of youth aged 11, 13, and 15 years [31]. Detailed information about the HBSC study methodology have been previously reported [31]. Internationally standardized and validated self-report questionnaires were administered in classrooms after instruction by a teacher or trained researcher. Adolescents’ self-report was anonymous. Active or passive consent from parents, youth, and school administrators were obtained prior to survey administration (following the specific requirements in different countries). Institutional ethics approval was obtained for each country. An amalgamated international dataset is made available for analysis.

### Measures

#### Bullying and Victimization

Four indicators of bullying involvement were measured (e.g., in-person bullying perpetration, in-person bullying victimization, cyber-bullying perpetration, cyber-victimization). In-person bullying perpetration and victimization were assessed using a modified version of the Olweus Bullying scale [32] with items asking how frequently the participants bullied another student (perpetration) or was bullied (victimization) at school in the past couple of months. Responses to in-person bullying perpetration and victimization items were dichotomized based on whether such behaviours occurred more than once or twice in the last 2 months (yes or no). Perpetration and victimization by cyberbullying were assessed via similar items but with responses dichotomized by presence or absence of any engagement in such behaviours. Prior to completing the questionnaire, participants were provided a definition of bullying that described different forms of bullying (e.g., verbal, relational), depicted the power differential existing in bullying dynamics, and distinguished bullying from other forms of conflict, such as teasing. Items that measured cyberbullying involvement provided specific examples of bullying behaviours, including sending mean electronic messages, and posting unflattering pictures without permission.

#### Mental Health

We examined two negative indicators of mental health status. Low life satisfaction was measured via the Cantril ladder which involves having adolescents indicate how satisfied they are with life using a simple analog scale ranging from 0 (worst possible life) to 10 (best possible life), with 5 or lower considered to be low [33]. Second, psychological symptoms were measured with a 4-item scale assessing the frequency of experiencing the following four psychological symptoms (feeling low, irritability or bad temper, feeling nervous, difficulty sleeping) over the past 6 months [34]. Items assessing psychological symptoms were selected from an original 8-item HBSC psychosomatic symptom checklist. Responses were scored on a 5-point scale (1 = Rarely or never, 2 = About every month, 3 = About every week, 4 = More than once a week, 5 = About every day). Sum scores were computed to classify participants as experiencing high psychological symptoms if they had a score of 12 or more, equivalent to an average rating of “about every week” or more frequent, across the four psychological symptoms.

#### Relationships With Supportive Adults

The presence of supportive adults in youths’ lives was assessed by focusing on the presence of supportive parent and teacher relationships. Parental support was assessed via the item “*My family really tries to help me*” from the Multidimensional Scale of Perceived Social Support (MSPSS; 1 = Strongly disagree to 7 = Strongly agree) [35]. A score of 5 or greater was taken to indicate the presence of a supportive parent. The presence of a supportive teacher support was indicated if the student agreed with the question “*I feel that my teachers care about me as a person*” [31]. Parent and teacher support were combined into a summary indicator of relationships with supportive adults with three levels: no supportive adults, either parent or teacher, or both parent and teacher.

#### Confounders

Other variables included gender (boy, girl), age group (11, 13, 15 years), and socio-economic status measured with the Family Affluence Scale-III (FAS-III). The FAS-III is a 6-item measure of material assets in the home including number of vehicles, bedroom sharing, computer ownership, bathrooms at home, dishwashers at home, and family vacations [36].

### Statistical Analysis

All analyses were conducted using SAS Version 9.4 [37]. Proportions of respondents reporting each of the indicators of bullying, mental health problems, and relationships with supportive adults were examined within and across the 45 countries and regions using conventional descriptive statistics. A series of multivariable Poisson regression models were used to estimate relative risks (RRs) and associated 95% confidence intervals (CIs) examining the associations between bullying involvement and mental health in each country. All analyses were stratified by gender and age group. The models adjusted for family affluence and those examining perpetration as a predictor were further adjusted for the equivalent victimization behaviour and vice versa. After conducting analyses within each of the targeted age groups, we limited presentation of findings to the oldest group (15 years) where the patterns were most illustrative. Clustering by school was adjusted for using Generalized Estimating Equations [38] and all analyses were weighted. To evaluate the consistency of effects across the 45 countries and regions, for each model we reported the number of countries where: 1) the RR = 1 (i.e., the 95% CI included a value of 1.00); 2) the RR >1 then 3) RR <1 (i.e., the CI did not include 1.00). Model convergence problems occurred among girls in four countries.

Finally, we examined whether having relationships with supportive adults moderated the associations between bullying and mental health problems, using the interaction term approach [39, 40]. Models were run in pooled analyses that combined data from all 45 countries and both available genders. The models accounted for two levels of clustering (students within schools then countries) and were adjusted for age, sex, family affluence, and bully victimization or perpetration depending on the model (note: adjustment for additional social factors did not change effect estimates). Model-based effect estimates were compared between the three strata defined by our supportive adult variable.

## Results

### Descriptive Results

In total, 230,757 students from 45 countries and regions were included in the analyses. The median sample size within participating countries was 4,520. There was a roughly equal number of boys (49.3%) and girls (50.7%) and students in each of the three age groups (11 years old = 33.8%; 13 years old = 34.5%; 15 years old 31.8%). The smallest sample was drawn from Greenland (*N* = 1,234) and the largest from Wales (*N* = 15,763).

Table 1 provides a description of the proportion of adolescents who reported bullying involvement and the two indicators of mental health problems. Proportions are presented using the median, minimum, and maximum values across all countries and regions. Among boys, bullying victimization and cybervictimization were more common than perpetration, and the prevalence of perpetration in general increased with age while the prevalence of bullying victimization and cybervictimization decreased. Girls on average reported less frequent bullying perpetration and victimization, and cyberbullying perpetration than boys, but equivalent frequencies of cybervictimization. The prevalence of mental health problems increased with age and were consistently worse among girls compared to boys.

**TABLE 1:** Frequency of bullying, cyber-bullying, high psychological symptoms, and low life satisfaction in 45 countries, by sex and age group (Health Behaviour of School Aged Children, International, 2017–2018).

Boys	Bully perpetrator	Bullying victim	Cyberbully perpetrator	Cyberbully victim	High psychological symptoms	Low life satisfaction
	Med% (min-max)	Med% (min-max)	Med% (min-max)	Med% (min-max)	Med% (min-max)	Med% (min-max)
Overall	6.5 (1.3–24.4)	9.6 (2.8–29.3)	10.4 (3.4–26.5)	12.2 (4.0–24.4)	21.3 (11.2–38.1)	10.0 (4.6–16.9)
Age 11	5.5 (0.4–20.6)	11.3 (5.1–29.3)	7.9 (1.8–21.2)	13.0 (3.9–26.9)	19.2 (9.5–36.5)	8.1 (2.4–16.6)
Age 13	6.6 (2.1–23.5)	10.7 (2.4–32.0)	10.5 (3.0–29.0)	12.0 (3.7–24.7)	21.4 (10.3–38.2)	9.8 (5.0–21.9)
Age 15	8.1 (1.9–29.6)	7.0 (1.6–26.1)	12.6 (3.7–31.1)	11.4 (3.2–28.7)	23.7 (11.5–39.6)	13.1 (4.7–21.8)
**Girls**						
Overall	2.9 (0.8–13.4)	8.5 (3.1–26.1)	6.7 (1.9–17.0)	12.1 (5.6–22.8)	33.5 (16.9–52.7)	14.5 (8.3–22.4)
Age 11	2.8 (0.3–12.4)	9.7 (4.4–26.4)	5.9 (0.7–15.6)	11.5 (3.6–24.3)	23.9 (10.8–37.5)	9.8 (3.6–17.1)
Age 13	3.3 (0.5–16.8)	8.7 (2.4–30.6)	7.5 (2.4–18.7)	13.5 (5.4–26.0)	35.0 (11.9–54.7)	15.7 (7.3–24.0)
Age 15	3.2 (0.5–14.8)	6.2 (2.1–21.3)	7.3 (1.5–19.1)	12.2 (5.2–22.0)	43.3 (21.2–65.5)	18.4 (9.7–29.5)

All values are weighted. Med% = Median proportion of the 45 countries. High psychological symptoms indicates a sum score of 12 or greater on a 4-item scale scored on a 5-point scale, equivalent to experiencing symptoms “about every week” or more frequent, across the four psychological symptoms. Low life satisfaction indicates a score of 5 or lower on a Cantril ladder ranging from 0 (worst possible life) to 10 (best possible life).

The vast majority of adolescents reported the presence of one or more supportive relationships with parents and teachers (Table 2). The proportion of youth reporting no supportive relationships with adults increased with age in both boys and girls, with analogous declines reported by age in those reporting supportive relationships with both parents and teachers.

**TABLE 2:** Relationships with supportive adults in 45 countries, by sex and age group (Health Behaviour of School Aged Children, International, 2017–2018).

	No supportive parent	No supportive teacher	Number of supportive adults		
			None	Parent or teacher	Parent and teacher
Boys	Med% (min-max)	Med% (min-max)	Med% (min-max)	Med% (min-max)	Med% (min-max)
Overall	13.9 (5.3–44.9)	36.9 (17.1–57.9)	6.5 (1.6–20.2)	38.5 (21.6–56.0)	53.7 (27.1–75.6)
Age 11	11.4 (4.0–41.6)	24.0 (9.9–47.9)	4.0 (0.90–18.4)	31.7 (13.3–49.4)	64.0 (32.2–85.3)
Age 13	14.3 (4.0–44.6)	39.9 (15.9–62.4)	6.7 (0.93–17.8)	39.3 (20.4–59.2)	52.8 (26.8–78.7)
Age 15	16.2 (5.8–49.1)	44.2 (20.8–61.8)	9.2 (2.2–24.7)	43.6 (26.1–58.5)	45.6 (21.8–70.0)
**Girls**					
Overall	15.3 (4.5–38.8)	37.7 (16.4–60.5)	7.6 (1.5–17.7)	37.0 (22.8–56.4)	53.7 (31.8–75.2)
Age 11	11.6 (3.3–39.3)	21.7 (8.2–47.0)	4.0 (0.28–17.5)	30.2 (11.4–47.6)	65.7 (35.0–86.9)
Age 13	15.0 (3.9–42.8)	42.9 (14.9–64.7)	9.3 (0–21.4)	40.2 (19.0–57.2)	50.5 (28.2–81.1)
Age 15	18.8 (5.7–44.0)	48.4 (14.5–76.2)	11.5 (2.7–25.1)	43.8 (22.2–66.7)	41.8 (22.3–75.1)

All values are weighted. Med% = Median proportion of the 45 countries.

### Relationships Between Bullying Involvement and Mental Health

Indicators of bullying involvement were consistently associated with increased risk of mental health problems across the 45 countries, although non-significant effects were not uncommon (Table 3). These effects are summarized visually for bullying perpetration and high psychological symptoms in 15-year-old boys (Supplementary Figure S1) and cyberbullying victimization and low life satisfaction in 15-year-old girls (Supplementary Figure S2). The negative effects of these different forms bullying involvement are clearly apparent in these forest plots.

**TABLE 3:** Number of countries (Total = 45) with a statistically significant association between bullying involvement and mental health indicators in 15-year-old boys and girls (Health Behaviour of School Aged Children, International, 2017–2018).

Predictor	High psychological symptoms			Low life satisfaction		
	Adjusted RR*			Adjusted RR*		
Boys – Age 15	RR>1	RR = 1	RR<1	RR>1	RR = 1	RR<1
Bully perpetrator	16	29	0	8	36	0
Bullying victim	31	14	0	21	23	0
Cyberbully perpetrator	17	28	0	10	35	0
Cyberbully victim	27	18	0	22	23	0
**Girls– Age 15**						
Bully perpetrator	14	31	0	4	40	0
Bullying victim	36	9	0	28	15	0
Cyberbully perpetrator	13	32	0	11	34	0
Cyberbully victim	39	6	0	34	11	0

RR, relative risk. All models are adjusted for family affluence, and models examining perpetration as a predictor are adjusted for victimization and vice versa. All models are adjusted for clustering by school and weighted depending on the country-specific approach to weighting. If the number of countries does not sum to 45 for a given predictor it is because the models did not converge in some countries. High psychological symptoms indicates a sum score of 12 or greater on a 4-item scale scored on a 5-point scale, equivalent to experiencing symptoms “about every week” or more frequent, across the four psychological symptoms. Low life satisfaction indicates a score of 5 or lower on a Cantril ladder ranging from 0 (worst possible life) to 10 (best possible life).

### Moderating Effects of Supportive Adults

Table 4 describes the proportion of youth reporting high psychological symptoms and low life satisfaction by the number of supportive adults in their life. As indicated by the *p*-trend, we found a statistically significant linear decrease in the proportion of students reporting mental health problems as the number of supportive adults increased from none to two (both parent and teacher), for boys and girls in all age groups.

**TABLE 4:** Description of mental health indicators in the full sample, by number of supportive adults (Health Behaviour of School Aged Children, International, 2017–2018).

	High psychological symptoms						*p*-trend
	None		Parent or teacher		Parent and teacher		
	%	(95% CI)	%	(95% CI)	%	(95% CI)	
Overall	50.4	(49.7–51.1)	32.5	(32.2–32.8)	21.3	(21.0–21.5)	<.001
Gender							
Boys	37.4	(36.4–38.5)	25.0	(24.5–25.4)	16.7	(16.4–17.1)	<.001
Girls	60.7	(59.7–61.6)	39.6	(39.2–40.1)	25.6	(25.3–26.0)	<.001
Age							
11	39.4	(37.8–41.1)	27.0	(26.4–27.6)	18.3	(17.9–18.6)	<.001
13	49.7	(48.5–51.0)	31.9	(31.3–32.5)	21.5	(21.1–21.9)	<.001
15	55.7	(54.7–56.8)	37.0	(36.4–37.5)	25.3	(24.8–25.8)	<.001
	**Low life satisfaction**						
	**None**		**Parent or Teacher**		**Parent and Teacher**		
	**%**	**(95% CI)**	**%**	**(95% CI)**	**%**	**(95% CI)**	
Overall	34.0	(33.3–34.7)	14.5	(14.2–14.7)	7.4	(7.3–7.6)	<.001
Gender							
Boys	26.5	(25.5–27.5)	11.7	(11.4–12.0)	6.3	(6.1–6.5)	<.001
Girls	39.9	(38.9–40.8)	17.1	(16.7–17.5)	8.5	(8.3–8.7)	<.001
Age							
11	28.0	(26.5–29.5)	12.1	(11.7–12.6)	6.1	(5.9–6.3)	<.001
13	34.1	(33.0–35.3)	14.0	(13.6–14.4)	7.5	(7.2–7.8)	<.001
15	36.5	(35.4–37.5)	16.6	(16.2–17.0)	9.2	(8.9–9.6)	<.001

High psychological symptoms indicates a sum score of 12 or greater on a 4-item scale scored on a 5-point scale, equivalent to experiencing symptoms “about every week” or more frequent, across the four psychological symptoms. Low life satisfaction indicates a score of 5 or lower on a Cantril ladder ranging from 0 (worst possible life) to 10 (best possible life).

Finally, Table 5 summarizes the pooled analysis examining effect modification of the associations between bullying and mental health by the presence of supportive adults. We found that risks for mental health difficulties (high psychological symptoms and low life satisfaction) associated with bullying involvement were greatest in students reporting multiple supportive adults. This trend was observed consistently for each of the four indicators of bullying involvement. We conducted additional analyses to examine the moderating effects of supportive parents and supportive teachers separately. Similar to the pooled analysis findings, we found the risks of poor mental health associated with bullying involvement were greater among youth reporting having a supportive parent compared to no supportive parent, and greater among youth reporting presence of a supportive teacher versus no supportive teacher. Furthermore, when we examined the effect modification of the associations across gender and age-groups, we found that the moderating effect of social support was consistent in boys and girls, and across the different age-groups.

**TABLE 5:** Associations between bullying involvement and mental health indicators in the full sample by number of supportive adults (Health Behaviour of School Aged Children, International, 2017–2018).

	High psychological symptoms					
	**None**		**Parent or teacher**		**Parent and teacher**	
Predictors	RR	(95% CI)	RR	(95% CI)	RR	(95% CI)
Bully perpetrator	1.09	(1.04–1.15)	1.21	(1.17–1.26)	1.40	(1.33–1.47)
Bullying victim	1.42	(1.37–1.47)	1.67	(1.62–1.71)	2.05	(1.98–2.12)
Cyberbully perpetrator	1.06	(1.02–1.10)	1.18	(1.15–1.22)	1.29	(1.24–1.35)
Cyberbully victim	1.37	(1.32–1.41)	1.57	(1.53–1.61)	1.84	(1.78–1.90)

**Predictors**	**Low life satisfaction**					
	**None**		**Parent or teacher**		**Parent and teacher**	
	**RR**	**(95% CI)**	**RR**	**(95% CI)**	**RR**	**(95% CI)**
Bully perpetrator	0.99	(0.92–1.07)	1.18	(1.11–1.26)	1.25	(1.13–1.37)
Bullying victim	1.50	(1.43–1.58)	1.96	(1.87–2.05)	2.44	(2.29–2.61)
Cyberbully perpetrator	0.98	(0.91–1.04)	1.04	(0.98–1.10)	1.18	(1.09–1.28)
Cyberbully victim	1.42	(1.35–1.50)	1.76	(1.68–1.85)	1.99	(1.86–2.13)

RR, relative risk. All models are adjusted for sex, age group, and family affluence, and models examining perpetration as a predictor are adjusted for victimization and vice versa. All models are adjusted for clustering by country and school, and weighted. High psychological symptoms indicates a sum score of 12 or greater on a 4-item scale scored on a 5-point scale, equivalent to experiencing symptoms “about every week” or more frequent, across the four psychological symptoms. Low life satisfaction indicates a score of 5 or lower on a Cantril ladder ranging from 0 (worst possible life) to 10 (best possible life).

## Discussion

The current study used recent data from the cross-national HBSC study to examine associations between bullying involvement (in-person and cyber) and mental health problems (psychological symptoms and low life satisfaction) in an international representative sample of adolescents. In line with initial hypotheses, we found that both in-person and cyber-bullying perpetration and victimization were consistently associated with higher levels of psychological symptoms and lower life satisfaction across the 45 countries and regions. Both in-person and cyber-bullying remain prevalent problems globally that negatively affect all youth involved. Our findings highlight that any form of bullying involvement is detrimental to youths’ mental wellbeing across various cultural contexts.

The current study also examined how supportive adults (e.g., parents and teachers) mitigate the mental health risks associated with bullying involvement. We found that in general, having supportive relationships with adults was related to better mental health outcomes. Results indicated there was a substantial decrease in the proportion of youth experiencing high psychological symptoms and low satisfaction as the number of supportive adults increased from none to having both a supportive parent and teacher. This finding is consistent with research suggesting that having caring, supportive adults in developmentally salient contexts (e.g., home and school) is important in promoting positive mental health amongst adolescents [22, 41].

While we originally hypothesized that having supportive relationships with adults would protect youth even further from the mental health risks associated with bullying involvement, we found that the opposite was true. Even though having supportive adults was associated with better mental health in general, we found that both in-person and online bullying perpetration and victimization were associated with increased risks of psychological symptoms and low life satisfaction among youth reporting more supportive adults in their lives. This pattern followed a dose-response relationship in the opposite direction of what was originally hypothesized. Mental health risks associated with bullying involvement increased as the number of supportive adults increased. This finding is perplexing, given that previous research has found that supportive teacher and parents mitigate the psychological harms related to bullying victimization and perpetration [19, 21]. In contrast, our findings suggest that having supportive adults exacerbate the negative mental health risks associated with both in-person and online bullying involvement.

One possible explanation of our findings is that perhaps adolescents perceive adults in their lives to be unhelpful in addressing bullying and cyber-bullying. Rigby and Bagshaw [42] found that less than 50% of adolescents perceived their teachers as helpful in addressing bullying and about 20% actually felt that interventions by teachers made the bullying worse. Other studies have found that different adult responses to bullying are perceived to be helpful to varying degrees [43, 44]. Despite reporting having supportive adults, it is possible that youth may perceive adults to be ineffective in addressing the bullying situation. Youths’ distress may be exacerbated if there are multiple adults unsuccessfully trying to intervene or if multiple adults provide unhelpful emotional support.

Second, it is possible that while youth report having supportive teachers and parents, youth do not seek help from them about bullying. When youth perceive that adults are unhelpful in addressing bullying, they are less likely to seek help [45]. Furthermore, as youth enter adolescence, seeking help from adults about bullying tends to decrease partly because youth perceive help-seeking to have detrimental consequences for their social standing and sense of autonomy [24]. Forming friendships that are mutually close and supportive is a central developmental task during adolescence [46]. Feedback and recognition from peers are particularly important at this developmental stage. As such, adolescents are more likely to seek support from their peers for bullying compared to adults [47]. One study found that when comparing peer support and family support, only peer support attenuated the negative effects of cyber-victimization on mental health [48]. Thus, despite having supportive adults in their lives, adolescents may not receive any help from adults because the bullying remains hidden or only known amongst peer groups. Perhaps youth with more supportive adults who are unaware of the bullying experience more distress because of increased feelings of helplessness and hopelessness in addressing the bullying dynamics.

Third, our findings may suggest that merely having supportive adults at home and school is not sufficient in protecting youth from experiencing the harms of bullying involvement. It is possible that even if adolescents do seek help from teachers and parents for their bullying experiences, these adults do not mitigate the risks associated with bullying. While help-seeking likely increases the likelihood that adults intervene in bullying situations, *how* adults intervene, and *how* adults support youth may be more relevant in mitigating the distress associated with bullying involvement. Troop-Gordon and Quenette [49] found that when teachers advocated for avoidance, independent coping, or assertion, peer victimization was associated with higher levels of internalizing problems. Dismissive or unhelpful advice from adults may increase feelings of helplessness and perpetuate self-blame among youth. Our findings highlight that just having relationships with more supportive adults do not necessarily protect youth from bullying-related distress. Rather, the presence of more supportive adults may exacerbate youths’ distress if bullying is not effectively addressed or if adults do not provide emotional support for youth experiencing bullying.

### Study Limitations and Future Directions

The current study was limited to measuring both teacher and parent support broadly using a single item for each adult because these specific items were consistently administered across all countries and regions. Our measure may not have been sensitive enough to assess how youth are supported specifically for issues related to bullying or cyber-bullying. Our measures did not assess whether adults were aware of the bullying and whether they have intervened in the past. Merely having a supportive parent or teacher may not be sufficient in addressing the complex emotional and psychological needs of youth who are involved in bullying. Future studies should measure *how* youth receive support from adults related to bullying, from a multidimensional framework that accounts for the different types of support that youth can receive (e.g., emotional support, instrumental support). Future studies should also examine how specific forms of support and adult responses to bullying moderate the relationship between bullying involvement and negative mental health. Studies should examine differences in adult support across developmental stages to provide clarity on adults who can protect youth involved in bullying throughout development.

The cross-sectional nature of this research also limits the interpretation of the directionality of the associations between bullying involvement and mental health indicators. Bullying involvement and mental health difficulties are related bidirectionally [9, 50]. There is a need to examine how supportive adults moderate the relationship between bullying involvement and mental health indicators longitudinally. Furthermore, level of adult support may be confounded with bullying severity. Adults may be more likely to get involved in more serious bullying situations and support the affected students [51]. Hence, our finding that more supportive adults exacerbate the adverse mental health problems related to bullying involvement could be attributed to the fact that high adult support is associated with more severe and more harmful bullying experiences. Longitudinal research is needed to untangle the relationship between bullying severity, adult support, and mental health problems. Future research can also clarify how supportive adults may prevent youth experiencing mental health difficulties from negative peer interactions that further perpetuate harm onto their mental wellbeing.

More research examining other protective factors that mitigate the mental health risks of bullying involvement is needed. In particular, the presence of supportive peers may be relevant in coping with bullying-related distress across cultural contexts. While parent and teacher support may be important during childhood, supportive peer relationships may be more relevant for adolescents, as peers become more salient in shaping youths’ attitudes and behaviours, and as youth develop heightened sensitivity to peer feedback [52]. As adolescents seek more autonomy from adults in their lives, peers may become increasingly more important as sources of emotional support. For adolescents, having supportive peers and being defended by peers during bullying situations may be particularly protective. Cross-cultural research examining how supportive peers may mitigate mental health difficulties related to bullying is needed.

The current research highlights both bullying and cyber-bullying to be prevalent experiences of adolescents worldwide. Involvement in bullying and cyber-bullying are related to low life satisfaction and higher levels of psychological symptoms. Contrary to our expectations, having supportive adults exacerbated the mental health risks associated with bullying involvement. Longitudinal studies are needed to examine how the impact of bullying involvement on youths’ mental health changes as a function of adult support.
