# Supplementary material for: Bullying, Mental Health, and the Moderating Role of Supportive Adults: A Cross-National Analysis of Adolescents in 45 Countries
Source: Int J Public Health. 2022 Mar 22;67:1604264. doi: 10.3389/ijph.2022.1604264 (PMC8980222; doi:10.3389/ijph.2022.1604264)

Figure S1
Associations between bully perpetration and high psychological symptoms in 15-year-old boys, by country (n= 45) (Health Behaviour of School Aged Children, International, 2017-2018)

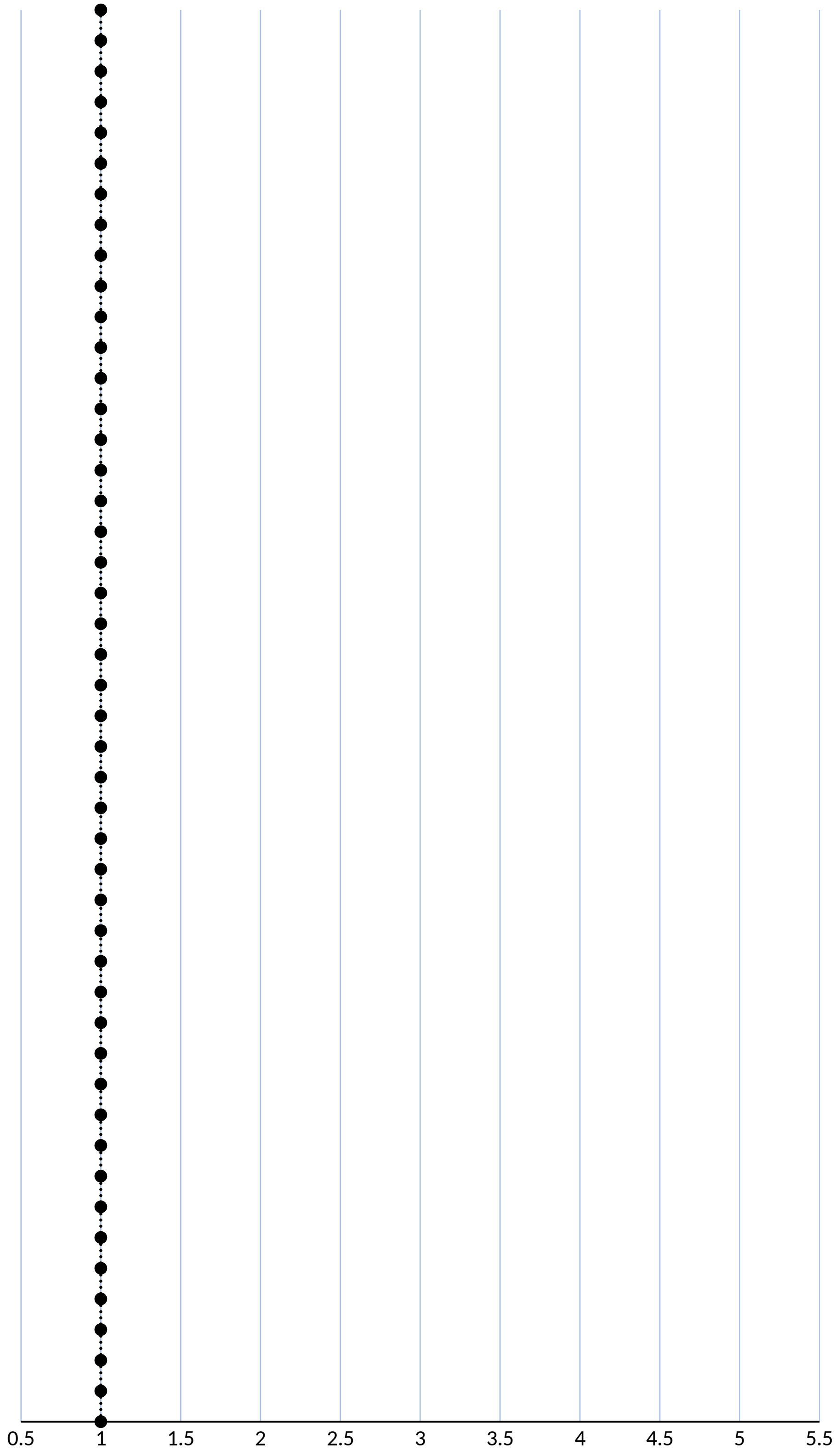

Figure S2
Associations between cyberbullying victimization and low life satisfaction in 15-year-old girls, by country (n= 45) (Health Behaviour of School Aged Children, International, 2017-2018)

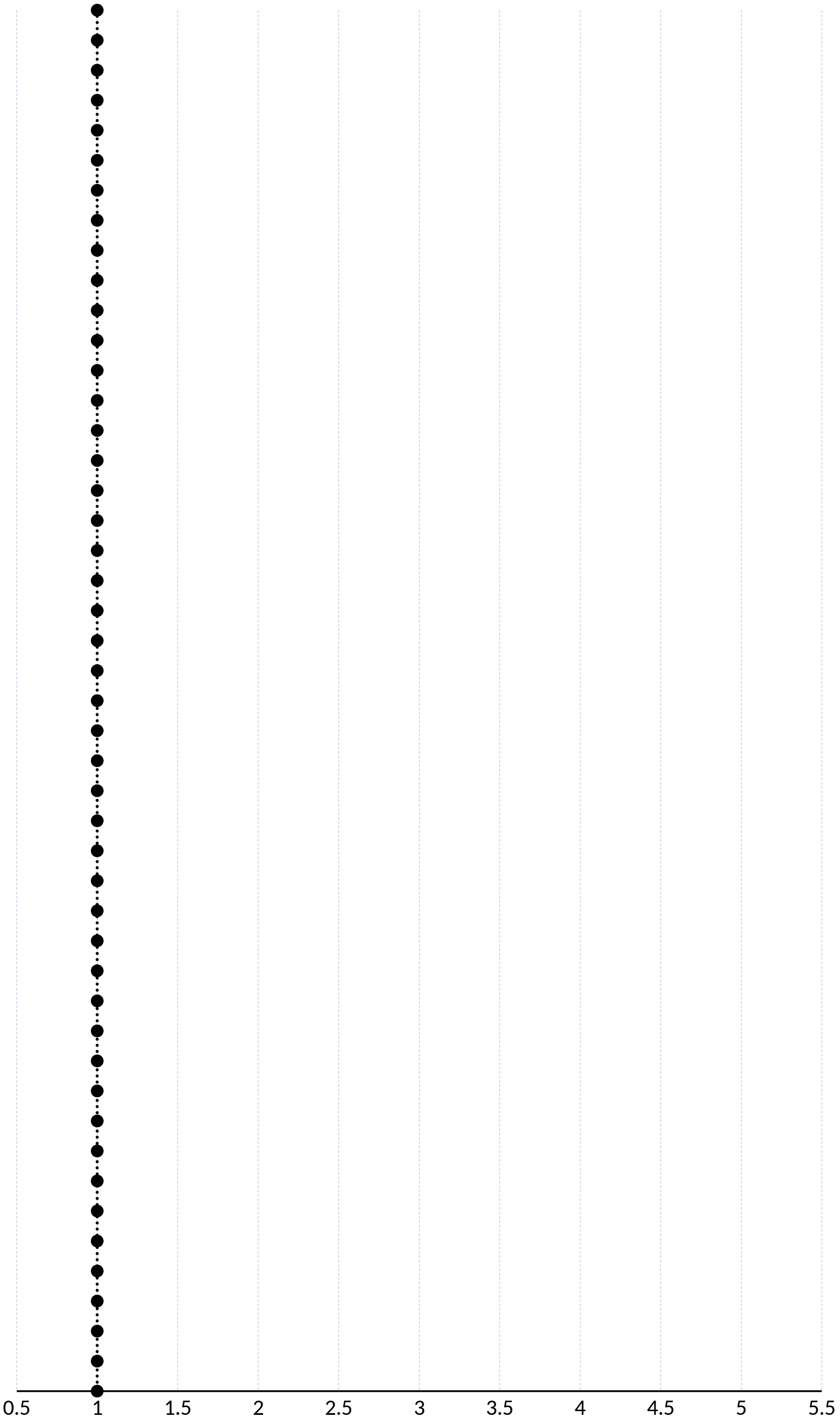

Supplement: Supplementary file 1 [file DataSheet1.docx]
